# Resistance training alters body composition in middle-aged women depending on menopause - A 20-week control trial

**DOI:** 10.1186/s12905-023-02671-y

**Published:** 2023-10-06

**Authors:** Eduard Isenmann, Dominik Kaluza, Tim Havers, Ana Elbeshausen, Stephan Geisler, Katharina Hofmann, Ulrich Flenker, Patrick Diel, Simon Gavanda

**Affiliations:** 1 Department of Fitness and Health, IST University of Applied Sciences, Düsseldorf, Germany; 2 Institute for Cardiovascular Research and Sports Medicine, German Sport University Cologne, Cologne, Germany

**Keywords:** Middle-aged women, Menopause, Hypertrophy, Muscle mass, Resistance training, Strength

## Abstract

**Background:**

Resistance training (RT) is effective in counteracting the age- and menopause-related loss of muscle mass (MM) and strength in middle-aged women (40–60 years). Research on RT with free weights is limited in pre- and post-menopausal women. Based on this, a 20-week training intervention was conducted with this population to investigate the effects of systematic RT with free weights on strength capacity and body composition.

**Method:**

Forty-one healthy women (52.0 ± 3.6 years) participated in this study. After 10-week control phase (no RT, T0-T1) followed by a 10-week intervention phase (T1-T2) with RT twice a week and 6–8 sets of each muscle per week. Subjects were randomly assigned to a low-intensity (50% 1-RM) or moderate-intensity (75% 1-RM) RT group and divided into pre-menopausal and post-menopausal according to their hormone profile. Fat-free mass (FFM), MM, fat mass (FM), muscle thickness (Vastus lateralis (VL), Rectus femoris (RF), Triceps brachii (TB)), grip strength, 1-RM squat and bench press were assessed before and after each phase. Statistical analysis was performed using a linear mixed model to account for fixed (time and group) and random (individual) effects.

**Results:**

A total of 31 women successfully completed the study. No injuries occurred during the intervention. Significant increases in 1-RM squat and bench press were observed in all groups. No interaction effect was observed for the strength parameters. In pre-menopausal women, FFM, MM and RF muscle thickness increased significantly, while VL showed a trend. These effects were not present in post-menopausal women regardless of RT intensity.

**Conclusion:**

RT with free weight is safe and effective for middle-aged women to increase 1-RM. Hypertrophy effects were found exclusively in pre-menopausal women. To achieve hypertrophy and/or body composition changes in post-menopausal women, larger training volumes (> 6–8 sets/muscle per week) are likely required.

**Supplementary Information:**

The online version contains supplementary material available at 10.1186/s12905-023-02671-y.

## Introduction

Loss of muscle mass (MM) is part of the ageing process [1]. MM in men and women has been shown to decrease by 3 to 8% per decade after the age of 30, and by 5 to 10% after age of 50 [2]. This reduction in MM and also strength during the ageing process may lead to physical disability [1], negatively affects the performance of everyday life, and increases the risks of falls and fractures [1, 3]. For context, post-menopausal women with reduced MM show a 2.1-fold higher risk of falling and a 2.7-fold higher risk of bone fracture than women with preserved MM [4]. Furthermore, since skeletal muscle is a highly metabolically active tissue, common metabolic disorders associated with ageing, such as diabetes, may also be associated with the decline in MM [1]. Therefore, maintaining MM during the ageing process is crucial for musculoskeletal health [3, 5, 6].

Probably the most significant event for ageing women is menopause, which usually occurs approximately between the ages of 45 and 55 [7]. Menopause marks the end of menstruation and reproductive capacity and is associated with various physiological hormonal changes. In particular, the decline in estrogen levels has detrimental effects on body composition, such as an increase in fat mass (FM), a decrease in MM, strength, and bone mineral density (BMD) [1, 8–11]. Consequently, menopausal women are at higher risk of developing osteoporosis and other musculoskeletal disorders [11, 12]. Hormone replacement therapy is feasible but is associated with side effects such as breast tenderness, enlargement, headaches, mood changes, or nausea. As an alternative or in addition to hormone replacement therapy, exercise interventions may be recommended [13]. Resistance training (RT) has been shown to be particularly effective in counteracting most of the negative effects of the menopause described above. There is very good evidence that progressive RT in older adults has positive effects on lean body mass [1, 14, 15], MM [16–18], strength [3, 16, 17], functional capacity [19, 20], bone mass and BMD [3, 14]. Moreover, it reduces risks of falls and fractures [21] and promotes physical and mental well-being [22], confidence and happiness [23].

Therefore, it is not surprising that the World Health Organization recommends that all adults should do muscle-strengthening activities that involve all major muscle groups at moderate or greater intensity and at least twice a week to provide health benefits [24, 25]. Recent reviews show that strength and muscle growth can be achieved at any intensity and number of repetitions [26, 27]. It seems that only 5–6 sets per muscle group per week are sufficient for beginners to induce adaptations [26, 27]. But current recommendations are based on data from males. Only 2–14% of the articles in three major sports and exercise magazines included only women as participants [28].

Previous studies employing middle-aged and older women primarily made use of machine-based programs or a combination of machine and free weight exercises [29, 30]. In addition, research has tended to focus on the effects of low to moderate-intensity RT programs (around 60% 1-RM) and 8–12 repetitions [1, 30, 31] but this is not consistent with current recommendations. The National Strength and Conditioning Association’s (NSCA) position statement on RT for older adults (i.e., > 50 years of age) specifically recommends RT with 1–3 sets per exercise per muscle group, two to three days per week with free weight or machine-based exercises using multi-joint movements at an intensity of 70–85% 1-RM including repetition ranges from 8 to 15 [32]. Usually, 15 or more repetitions are completed at an intensity of 60%, or an intensity of 70–80% is generally used for a repetition range of 8–12 repetitions [26, 27]. Besides, only a few studies compared the effects of different intensities in machine-based RT programs [33, 34, 35, 36]. Moreover, “effort”, or the set endpoint and exercise velocity was rarely described [29].

However, to increase strength, free weight training might be superior to machine-based programs [29, 32, 37]. In addition, movements of daily living can be ideally trained with free weights and are more akin to applied science [32]. It is therefore surprising that, to the best of our knowledge, no studies involving middle-aged or older women have been conducted exclusively with free weights. In summary, there is insufficient evidence to provide specific guidelines for older women, including pre-, peri-, and post-menopausal women, to optimize MM and strength gains [30]. Furthermore, participants’ hormonal status was not or insufficiently assessed prior to enrollment [38], and there is a lack of data on the effects of free weight RT in middle-aged women.

Therefore, this is the first study to investigate the effects of free weight RT on muscle strength and body composition in middle-aged women depending on hormonal status (pre- and post-menopausal) and two different intensities. This will include identifying potential differences in the development of fat-free mass (FFM), MM, and strength capacity, as well as FM reduction, according to pre- and post-menopausal status.

## Methods

### Participants

To determine the sample size, a power analysis (F-tests, Anova: Fixed effects, special, main effects and interaction) was performed a priori. For the calculation, a medium to strong effect (f) (0.25–0.40), an α-error of 0.05, and a power of 0.8 (1-β error) were specified. Based on the three-arm model (df = 2), a total sample size of 18–36 subjects was calculated. However, since the study will last for a total of 20 weeks and drop-outs were taken into account, the number of subjects was set at a minimum of 40. All volunteers had to be healthy, with no orthopedic or cardiovascular complaints and should be able to perform a squat with the tops of their thighs parallel to the floor. As a result, seven individuals were excluded before the start of the study. After being informed about the study procedures and inclusion criteria, 41 healthy women were enrolled in the study (15/03/2021) after signing informed consent. The classification of the participants as pre-menopausal and post-menopausal was based on hormone concentrations and the date of the last menstrual period [38]. Participants were classified as post-menopausal if they had low estradiol (E2) and high follicle-stimulating hormone (FSH) and had not menstruated for at least 12 months [38]. Seventeen participants (n = 17) were classified as pre- (PreMeno) and 24 as post-menopausal (PostMeno). Subsequently, PostMeno women were allocated to two subgroups after stratified randomization (MM, age, weight and height): moderate-intensity (MI-RT; n = 12) and low-intensity (LI-RT; n = 12). PreMeno women were not subdivided due to the small sample size and performed MI-RT.

### Experimental design

**Fig. 1:**
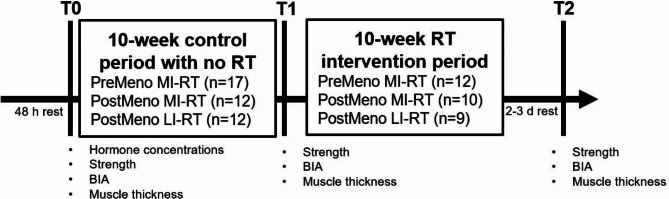
Schematic representation of study design. RT = resistance training; BIA = bioelectrical impedance analysis; PreMeno = pre-menopause; PostMeno = post-menopausal; MI-RT = moderate-intensity resistance training; LI-RT = low-intensity-resistance training

The study was approved by the local ethics committee of the IST University of Applied Science, Dusseldorf (02/2021), according to the Declaration of Helsinki, and registered in the German Registry of Clinical Studies (05/03/2021; DRKS00023826). In addition, the hygiene concept to prevent the spread of COVID-19 was approved by the local regulatory authority. A total of 41 women aged 40–60 years were recruited into a local gym. The study design encompassed two phases, and three measurement points (T0, T1, T2), and lasted for 20 weeks (see Fig. 1).

Initially, ten weeks (T0-T1) served as a control period without any training or systematic physical activity to examine the effects of reduced physical activity (gym lockdown due to the COVID-19 pandemic) on strength capacity and body composition. This was followed by a 10-week RT intervention period (T1-T2) using a two-group matched pair parallel design. At T0, participants completed questionnaires about their health, menstrual status, and recent RT history. Subsequently, hormone status (E2, progesterone (P), (FSH), testosterone (T), dehydroepiandrosterone (DHEA)), body composition (total body water (TBW), FFM, MM, FM), muscle thickness (vastus lateralis (VL), rectus femoris (RF), triceps brachii (TB)), grip strength (GS), and dynamic strength (1-RM squat (SQ), 1-RM bench press (BP)) were assessed. The same variables were also collected after the control and RT period except for hormone status. T2 testing was done 48–72 h after the last RT session.

### Procedures

Forty-eight hours before testing, the subjects were not allowed to engage in RT or any other strenuous physical activity. The volunteers were also not allowed to drink alcohol or coffee before testing and had to appear in a fasted state. However, about 300-400ml of water had to be drunk in the morning to equalize the water balance. All measurements (T0–T2) were conducted single-blind in the morning (7.30–11.00 am) at the same time of the day by the same researcher.

### Hormone parameters

The saliva and blood samples were collected immediately at the beginning of T0 (7.30-9.00am). In premenopausal women, saliva and blood samples were collected during the luteal phase (second half of the cycle), when both E2 and P concentrations are high. Hormone concentrations were used only for identification and classification between pre- and post-menopausal states. For saliva samples, specific ELISA kits for E2, P, T, and DHEA concentrations were used (RE52281; RE62141; RE52651; RE30121046; Re62039). Dry blood concentration of FSH was analyzed by an external laboratory according to the CLIA method (Ayumetrix, 17,387 63rd Ave, Lake Oswego, OR 97,035, USA).

### Body mass and body composition

Body weight (BW) and body composition were measured immediately after collecting salvia and blood samples. BW was assessed using a digital scale (Etekcity EB4074C, Anaheim, CA, United States of America), with participants wearing only underwear and no shoes or socks. TBW, FFM, MM, and FM were analyzed by bioelectrical impedance analysis (BIA 101, Akern, Firenze, Italy). BIA 101 Akern is a valid and reliable alternative method to dual-energy X-ray absorptiometry (DXA) for verification of body composition [39, 40]. BIA was performed using an alternating sinusoidal electric current of 400 microampere at an operating frequency of 50 kHz. For the bioelectrical impedance measurement, each participant was supine with limbs slightly spread apart from the body for 10 min to allow for fluid shift [41]. Disposable tab electrodes (BIATRODES Akern Srl; Florence, Italy) were placed on the right side at metacarpal and metatarsal sites of the right wrist and ankle [39]. Subsequently, the measurement was performed, and the data were processed using BodyGramPro software (Version 3.0, Akern, Firenze, Italy). Further information about the BIA 101 can be obtained from the manufacturer’s manual [42].

### Muscle thickness

For muscle thickness measurements, a B-mode ultrasound (Mindray DP-50, Mindray Medical International Ltd, Shenzhen, China) with an 8.5-MHz linear probe (Mindray 75L53EA, Mindray Medical International Ltd, Shenzhen, China) was used. Muscle thickness was measured at three sites in the muscles on the right side, in accordance with previous studies [41, 43].

M. vastus lateralis thickness was measured with the participants lying on their left side on an examination table at half the distance between the most prominent point of the greater trochanter and the lateral condyle of the tibia (gain 50 dB; image depth 3.7 cm). The thickness of the rectus femoris was measured at 50% between the anterior inferior supra iliac crest and the proximal border of the patella with participants lying supine (gain 50 dB; image depth 3.7 cm).

For measurement of the triceps brachii, the participants lay in the prone position while images were taken at 40% distal between the acromial process of the scapula and the lateral epicondyle of the humerus (gain 50 dB; image depth 5.5 cm). To ensure the identical positioning of the ultrasound probe, the measuring points were marked with a waterproof pen. Ultrasound transmission gel was applied to the probe head and the probe was positioned perpendicular to the long axis of the extremity without depression of the underlying tissue. Three images were recorded at each site and stored on a USB flash drive. Subsequently, muscle thickness was analyzed in the images using the caliper measurement of the ultrasound device. The mean values of the three images of each site were used for further analyses. The test–retest intraclass correlations coefficient for this analysis was reported from our laboratory as 0.998 (RF), 0.996 (VL), and 0.997 (TB) [41].

### Maximum strength tests

Following a standardized warm-up procedure (5 min running), grip, upper and lower body strength tests were conducted. Grip strength of both hands was assessed using a digital hand-held dynamometer (digital Jamar+, Fabrication Enterprises, New York, United States). For testing, volunteers were seated upright on a chair with their elbows bent at 90° and in contact with the body. Then they were instructed to press the handle of the device as forcefully as possible for at least five seconds without changing their position. Three trials were carried out for each side. Maximum strength was measured alternately. The rest period between repetitions on each side was 120 s. The best trial was documented for further analysis. For testing lower body strength, a “touch and go” barbell box squat (femur parallel to the floor, 90° knee angle) was used. The height of the box was individually adjusted for each subject and maintained throughout the RT period and during retesting. After a minimum of five minutes of rest, upper body strength was assessed using the free weight BP exercise. For this, grip widths were documented and stipulated throughout the study. For both tests, the participants first completed ten repetitions with an empty bar, followed by a two-minute rest period. A second warm-up set of ten repetitions was then performed with at approximately 50% of the predicted ten-repetition maximum load. Following four-minute rest, a final set was performed to the point of momentary concentric muscle failure or failure of proper exercise technique. This was done by pre-setting a load that the research team estimated would allow one to ten repetitions. Both exercises are well-established exercises for determining strength ability and upper and lower body performance [44]. From the load used and the number of repetitions completed, the 1-RM SQ and BP were calculated according to the formula proposed by Brzycki [45], which was considered sufficiently accurate for estimating 1-RM using fatiguing sets of less than ten repetitions [46].

### Resistance Training Protocol

A detailed description of the RT protocol (sets, repetitions, intensity, tempo, and rest) throughout the study can be found in Table 1. The training program consisted of two cycles of five weeks each. Weeks one to four of each cycle were “loading” weeks, followed by one “deload” week. Deload weeks were introduced to counteract possible over reaching due to too rapid increases training weights. Training was performed twice weekly, 48–72 h apart. All RT sessions were supervised by a qualified member of the research team (researcher-to-participant ratio 1:1–4). Exercise selection was identical for all intervention groups. Session 1 consisted of “touch and go” barbell box squats (femur parallel to the floor), barbell bench press, seated neutral grip cable row, dumbbell side bend, and prone plank. In the second training session, the same exercises were repeated, except that the cable row was replaced by a lat pull-down with a wide pronated grip. Except for the box squats and the plank, all exercises were performed with the maximum range of motion possible and at identical tempo. Volume loads (repetitions x sets x % 1-RM) were approximately similar between the intervention groups, with the MI-RT group performing more sets per exercise to achieve a similar volume load compared to the LI-RT group. The adjusted weight of the box squat and bench press in the first cycle was based on the initial 1-RM test (T1), whereas the resistance of the remaining exercises was determined by trial and error. During the “loading” weeks, the last set of each exercise was performed to momentary concentric failure or failure of proper exercise technique. In the following weeks, the weight for each exercise was increased by 2.5-5%, depending on the number of repetitions to failure. From the last set of each exercise in week 4, a new 1-RM was estimated using the Brzycki formula [45] and used from week six.

**Table 1:** Overview of the resistance training protocol

	Cycle 1		Cycle 2	
	Week1–4 Loading	Week5 Deload	Week6–9 Loading	Week10 Deload
	**Session 1 and 2** (sets x reps)			
**MI-RT** Intensity (% 1-RM)	**4sets** 3 × 10 1x to failure 75 ^1)^	**3sets** 3 × 10 53.3^1)^	**4sets** 3 × 10 1x to failure 75²^)^	**3sets** 3 × 10 53.3 ²^)^
**LI-RT** Intensity (% 1-RM)	**3sets** 2 × 20 1x to failure 50^1)^	**2sets** 2 × 20 40^1)^	**3sets** 2 × 20 1x to failure 50²^)^	**2sets** 2 × 20 40²^)^
**Tempo (s)** **Rest (s)**	2:0:1 (eccentric : isometric : concentric) 120 s			

*MI-RT = moderate-intensity resistance training group; LI-RT = low-intensity resistance training; 1-RM = one-repetition maximum;* ^*1)*^ *based on pre intervention 1-RM; ²* ^*)*^ *based on estimated 1-RM using the Brzycki formula (35) (weight and repetitions from the last set of each exercise following week 4 session 2)*

### Nutrition

Dietary habits were maintained throughout the 20 weeks. Participants were unfamiliar with comprehensive nutrition documentation and related tools, so it was not possible to establish it as standard practice from the outset. Further, due to the COVID-19 pandemic, it was not possible to provide more comprehensive and specific nutritional recommendations. Only immediately after the RT sessions was the diet standardized. Participants consumed a carbohydrate-protein source and could choose between three different carbohydrate and protein rich meals. Each meal has been used in previous investigations [47, 48]. Detailed meal information is provided in the supplementary material.

### Statistical analyses

Data were analyzed by the R statistical language version 4.0.4 (R Core Team, 2021). Only data from participants with an adherence of > 85% were included in the analyses. The raw data of VL, RF, and TB were box-cox transformed prior to analysis. The transformation was applied to obtain approximately Gaussian distributions of the raw data, which otherwise exhibited highly skewed distributions. Transformations were applied to the data at T_0_ and the box-cox estimates for λ were then employed to transform the remaining data.

The values of FFM, MM, FM, GS, 1-RM SQ and BP were analyzed unchanged. Individual time intervals (∆t [h]) since the start of the study were introduced as an additional covariate. Data analysis was performed using linear mixed effects (LME) models with FFM_λ_, MM_λ_, FM_λ_, VL_λ_, RF_λ_, TB_λ_, GS, 1RM SQ and BP used as dependent variables. Model building was performed independently for each of these.

We were specifically interested in the effects of menopause on the trends of strength capacity and muscle growth. Therefore, all models included the interaction term of menopause with PreMeno MI-RT (T0 to T2) as a fixed effect. Likewise, PreMeno MI-RT itself was axiomatically included as a fixed effect. As it represents an ordered factor with three levels, second-order orthogonal polynomials were chosen as contrasts for PreMeno MI-RT.

Initially, random effects were merely assumed between the individual intercepts of each measure. Subsequently, ∆t was included as a random effect, where linear individual trends were assumed. The presence of potentially non-linear individual trends was then investigated by upgrading to 2nd or 3rd-order natural splines of ∆t. After developing appropriate random effect structures, it was tested whether ∆t also contributed to general trends in the population, i.e., whether it represented a significant fixed effect. In either case, model comparisons were based on likelihood statistics and changes in Akaike’s information criterion. Significant differences were set at p ≤ .05.

Finally, effect sizes between discrete time levels were determined according to the approximation of Cohen’s *d* for mixed effects models (*d* = 2t/DF^(1/2)^) where t = t-value; DF = degrees of freedom. Classifications were stipulated as follows: trivial < 0.2; small < 0.5; moderate < 0.8; strong > 0.8 [49]. All graphs were created using the latest version of GraphPad Prism.

## Results

Five participants missed T1 testing and were therefore excluded from the second part of the study. Another five participants missed more than three RT sessions during the intervention period (T1–T2) and were therefore also excluded from the final analysis. The reasons for absence were non-study-related injury or illness (n = 4) and other personal reasons (n = 6). No injuries occurred during the RT intervention. In total, 31 subjects completed the study. The PreMeno women (n = 12) had an average age of 47.4 ± 5.3 years and a height of 167.5 ± 8.4 cm. The PostMeno MI-RT group (n = 10) had an average age of 54.3 ± 4.7 years and a height of 166.0 ± 7.2 cm. The anthropometric data for the PostMeno LI-RT group (n = 9) were 55.6 ± 2.9 years and 166.6 ± 5.9 cm. The hormone concentrations of the three training groups at T0 are shown in Table 2.

**Table 2:** Hormone concentration

	PreMeno MI-RT	PostMeno MI-RT	PostMeno LI-RT
E2 (pg/ml)	7.4 ± 17.1	1.1 ± 1.0	1.5 ± 1.2
P (pg/ml)	58.4 ± 40.2	51.2 ± 24.5	30.3 ± 11.8
FSH (mlU/ml)	16.7 ± 19.0	86.2 ± 33.9	96.5 ± 16.7
T (pg/ml)	14.4 ± 6.4	15.7 ± 7.9	16.0 ± 8.2
DHEA (pg/ml)	170.9 ± 72.0	195.6 ± 90.6	179.9 ± 77.9

*PreMeno = pre-menopause; PostMeno = post-menopause; MI-RT = moderate-intensity resistance training; LI-RT = low-intensity resistance training;DHEA = dehydroepiandrosterone; E2 = estradiol; FSH = follicle-stimulating hormone; n.a. = no analyses; P = progesterone; T = testosterone*

### Body composition

BW and body mass index (BMI) did not change significantly in none of the three groups during the entire period (BW: *p* = .494; BMI: *p* = .559). No difference between the groups could be determined for the BW (PostMeno MI-RT: *p* = .992; PostMeno LI-RT: *p* = .131) and BMI (PostMeno MI-RT: *p* = .503; PostMeno LI-RT: *p* = .115).

**Fig. 2:**
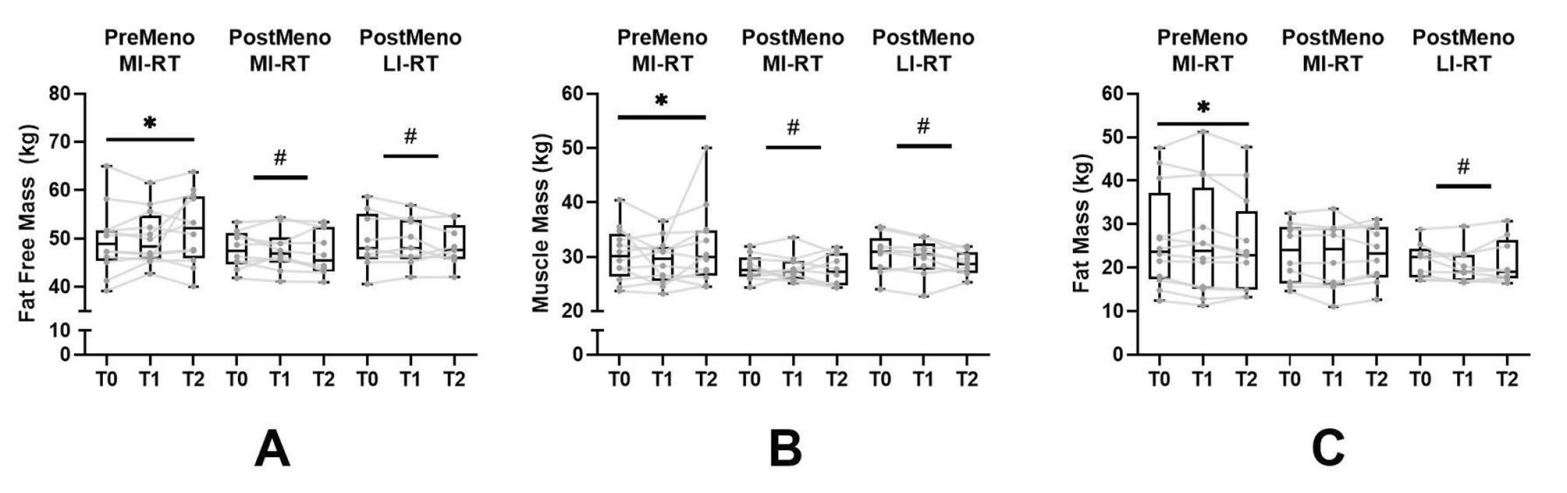
Body composition: fat-free mass, muscle mass, fat mass. Significant time and time*group effects were set p < .05. Time effects were marked with * and group effects with #

For FFM, only a significant increase was observed in the PreMeno MI-RT group (*p* = .015) (first order). The effect between T1 and T2 in the PreMeno MI-RT group was small (*d* = 0.29). In addition, an interaction effect (first order) of both PostMeno and PreMeno MI-RT could be identified (PostMeno MI-RT: *p* = .032; PostMeno LI-RT: *p* = .022). Unlike the PreMeno MI-RT group, the two PostMeno groups showed no increase in FFM (Fig. 2a).

In MM, only a significant increase was observed in the PreMeno MI-RT group (*p* = .002) (second order). The effect between T1 and T2 in the PreMeno MI-RT group was strong (*d* = 1.25). Additionally, the group effect (second order) of both PostMeno and PreMeno MI-RT could be identified (PostMeno LI-RT: *p* = .030; PostMeno LI-RT: *p* = .024). Unlike the PreMeno MI-RT group, the two PostMeno groups did not have an increase in MM (Fig. 2b).

In FM, only a significant decrease was detected in the PreMeno MI-RT group (*p* = .039) (first order). The effect between T1 and T2 in the PreMeno MI-RT group was moderate (*d* = 0.57). A Time*group interaction was only between the PostMeno LI-RT to the PreMeno MI-RT group (*p* = .039) (second order). In contrast to the PreMeno MI-RT group both PostMeno groups had no decrease in FM (Fig. 2C).

### Muscle thickness

**Fig. 3:**
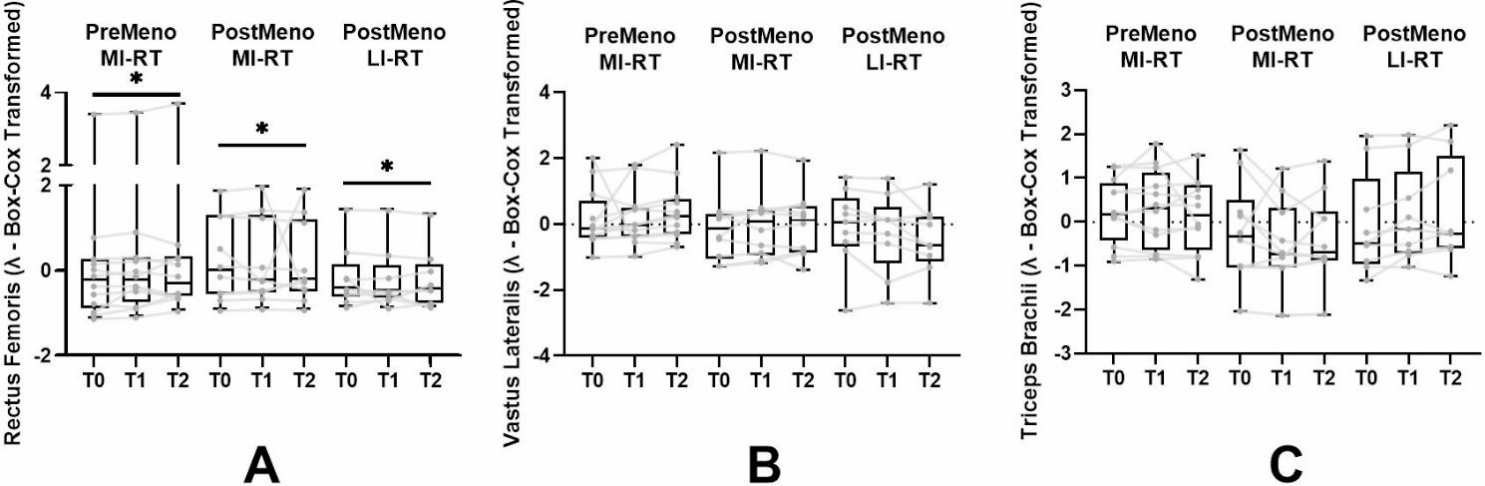
Muscle thickness of m. rectus femoris, m. vastus lateralis, m. triceps brachii. Significant time and time*group effects were set p < .05. Time effects were marked with * and time*group effects with #

A significant difference over time was observed in the RF (*p* = .004) (first order). Different curve progression could not be detected (PostMeno MI-RT: *p* = .210; PostMeno LI-RT: *p* = .300) (first order). The effect between T1 and T2 in all groups was moderate to strong (PreMeno MI-RT: *d* = 0.80; PostMeno MI-RT: *d* = 0.46; PostMeno LI-RT: *d* = 0.52) (Fig. 3a).

Trends over time could be identified in the VL (first order: *p* = .050; second order: *p* = .079). In the PreMeno MI-RT and PostMeno MI-RT groups, a small to moderate effect was found between T1 and T2 (PreMeno MI-RT: *d* = 0.54; PostMeno MI-RT: *d* = 0.43). In addition, a trend in the curvature could be observed between the PreMeno MI-RT and the PostMeno LI-RT group (*p* = .058) (first order) (Fig. 3b).

In the TB, no significant difference over time could be detected (first order: *p* = .72; second order: *p* = .83). In addition, no group differences were observed (Fig. 3c).

### Strength

**Fig. 4:**
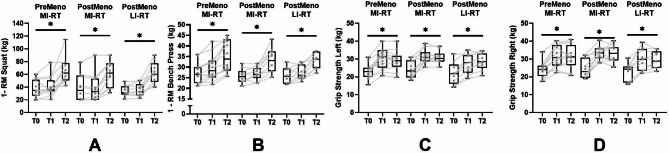
Strength parameter: 1-RM squat, 1-RM bench press, grip strength. Significant time and time*group effects were set at p < .05. Time effects were marked with * and time*group effects with #

All three groups increased their SQ performance significantly over time (first order: *p* = .000 and second order: *p* = .002). No group differences were observed in SQ performance (PostMeno MI-RT: *p* = .257; PostMeno LI-RT: *p* = .913 first order) (Fig. 4A). A strong effect was detected over time for all groups (PreMeno MI-RT: *d* = 1.51; PostMeno MI-RT: *d* = 1.52; PostMeno LI-RT: *d* = 1.64 s order). Each group improved significantly in BP performance (first order: *p* = .000 and second order: *p* = .000) (Fig. 4B). No significant difference in curve progressions could be observed between the groups. (PostMeno MI-RT: *p* = .712; PostMeno LI-RT: *p* = 795, first order). A moderate to strong effect over time was detected for each group (PreMeno MI-RT: *d* = 0.66; PostMeno MI-RT: *d* = 0.98; PostMeno LI-RT: *d* = 0.84 second order). In grip strength, an identical development was found for the left and right hand and was summarized as one score. Both the first and second-order terms were significant (first order: *p* = .000 second order: *p* = .000) (Fig. 4C&D). No differences were detected between the three groups (PostMeno MI-RT: *p* = .548; PostMeno LI-RT: *p* = .675, first order).

All results are summarized in Table 3.

**Table 3:** all results of body composition, muscle thickness and strength capacity

	PreMeno MI-RT			PostMeno MI-RT			PostMeno LI-RT		
	T0	T1	T2	T0	T1	T2	T0	T1	T2
Age (y)	47.4 ± 5.3			54.3 ± 4.7			55.6 ± 2.9		
Height (cm)	167.50 ± 7.99			166.00 ± 6.78			166.56 ± 5.58		
BW (kg)	76.19 ± 16 0.31	76.18 ± 16.18	75.62 ± 16.54	70.29 ± 8.61	70.43 ± 8.83	70.04 ± 8.33	71.10 ± 8.52	69.68 ± 7.90	69.56 ± 8.35
BMI (kg/m^2^)	27.28 ± 5.78	27.25 ± 5.95	27.03 ± 5.82	25.81 ± 2.15	25.52 ± 2.48	25.37 ± 2.26	25.62 ± 2.79	25.06 ± 2.90	25.12 ± 3.06
FFM (kg)	**49.58 ± 6.78**	**50.08 ± 5.41**	51.97 ± 6.99*	**47.70 ± 3.63**	**48.07 ± 3.89**	**46.99 ± 4.41#**	**49.49 ± 5.47**	**49.20 ± 4.61**	**48.46 ± 4.00#**
MM (kg)	**30.48 ± 4.77**	**29.18 ± 3.95**	31.88 ± 7.05*	**27.98 ± 2.22**	**27.78 ± 2.34**	**27.59 ± 2.74#**	**30.56 ± 3.47**	**29.80 ± 3.24**	**28.77 ± 2.09#**
FM (kg)	**26.44 ± 11.13**	**26.09 ± 12.22**	24.82 ± 10.77*	23.49 ± 6.40	22.84 ± 7.25	23.10 ± 6.07	**21.61 ± 3.74**	**20.48 ± 3.93**	**21.32 ± 4.85#**
VL (cm)	**2.39 ± 0.42**	**2.40 ± 0.45**	2.62 ± 0.48*	**2.25 ± 0.48**	**2.29 ± 0.53**	2.41 ± 0.51*	**2.26 ± 0.56**	**2.15 ± 0.62**	**2.15 ± 0.55§**
RF (cm)	**1.95 ± 0.85**	**1.98 ± 0.84**	2.18 ± 0.85*	**2.13 ± 0.66**	**2.07 ± 0.68**	2.23 ± 0.66*	**1.84 ± 0.48**	**1.77 ± 0.49**	1.95 ± 0.47*
TB (cm)	3.29 ± 0.39	3.29 ± 0.47	3.26 ± 0.46	3.01 ± 0.58	2.87 ± 0.50	2.96 ± 0.54	3.11 ± 0.60	3.20 ± 0.56	3.31 ± 0.66
GS left (kg)	**23.12 ± 4.16**	30.32 ± 5.51*	29.16 ± 5.26	**24.16 ± 4.53**	31.52 ± 3.78*	30.96 ± 3.46	**21.99 ± 5.84**	27.20 ± 5.11*	28.74 ± 4.39
GS right (kg)	**23.95 ± 4.28**	31.45 ± 5.72*	31.27 ± 6.23	**24.59 ± 5.01**	33.30 ± 3.61*	33.09 ± 4.48	**22.68 ± 4.76**	29.80 ± 5.85*	29.76 ± 4.46
1-RM SQ (kg	**37.91 ± 12.99**	**40.85 ± 14.83**	65.80 ± 18.76*	**39.01 ± 19.64**	**36.83 ± 21.45**	60.71 ± 18.91*	**34.27 ± 8.07**	**35.49 ± 9.20**	62.77 ± 15.87*
1-RM BP (kg)	**27.24 ± 4.86**	**29.77 ± 5.92**	35.12 ± 7.10*	**25.83 ± 3.19**	**27.01 ± 2.95**	33.05 ± 5.45*	**25.98 ± 3.58**	**27.47 ± 3.18**	33.38 ± 3.62*

*PreMeno = pre-menopause; PostMeno = post-menopause; MI-RT = moderate-intensity resistance training; LI-RT = low-intensity resistance training; FFM = fat-free mass; FM = fat mass; MM = muscle mass; VL = vastus lateralis; RF = rectus femoris; TB = triceps brachii; GS = grip strength; 1-RM SQ = one repetition maximum squat; 1-RM BP = one repetition maximum bench press; Significant time effects were set at p* ≤ .05 and were marked with *. If no difference was identified between the groups over time, this was assumed for all groups. Time*group differences were marked with #. The Premeno MI-RT group was used as a control treatment and the curves of the two post-menopausal groups were compared. Trends (p < .10) showing potential statistical differences between two groups and were marked with §

## Discussion

This study investigated, the effectiveness of systematic RT using free weight on strength, and body composition in middle-aged women (40–60 years). For this purpose, a 20-week intervention with a 10-week control-phase and a 10-week training phase was conducted. The results show different effects on body composition for PreMeno and PostMeno women, but not on strength gains. PreMeno women with higher E2 concentrations and an active menstrual cycle significantly increased their FFM (small effect), MM (strong effect) and muscle thickness in VL (moderate effect) compared to PostMeno women regardless of training intensity. However, similar increases in lower and upper body strength as well as grip strength were achieved in all groups. In contrast to the hypertrophy effects, there appear to be no differences depending on hormone concentrations.

In general, there is a lack of studies on RT in middle-aged women using free weights only. However, flexion and extension movements in the hip joint, as well as traction and compression stresses on the shoulder joint, are everyday strains for active individuals. Free weight exercises such as squats and bench presses, which can simulate movements of daily living, may be ideal for increasing strength at a high level of specificity [29, 37, 50]. There are currently no studies comparing the effects of free-weight RT alone with machine-based interventions in PreMeno or PostMeno women. However, the present study has shown that free weight RT can be used safely and effectively in middle-aged women. In addition, our results demonstrate that low and moderate-intensity RT using free weights is effective for increasing strength in PreMeno and PostMeno women, both in the upper and lower body. These findings corroborated observations from previous studies based on younger participants [37, 51, 52]. For example, Botero and colleagues showed that three RT sessions per week over 12 months could increase BP and leg press performance [53]. The strength increases in our study are comparable to the effects reported in previous research [54, 55]. In comparison, Karaslaan and colleagues showed a stronger effect with 4 training sessions/week for 12 weeks with machine-assisted training [56]. Similar to the male participants, the studies on middle-aged women show a comparable dose-response relationship in the adaptation processes of muscular strength [57]. Interestingly, this study did not find any differences between PreMeno and PostMeno women. The change in endocrine homeostasis probably has no significant effect on strength capacity in untrained healthy middle-aged women. In this population, other factors such as neuronal activation presumably could play an important role in the first few weeks of RT. At present, however, there is no indication of how many training experiences are needed to rule out strong neural adaptations and rather attribute the effects to endocrine homeostasis for strength adaptations. Therefore, the influence of endocrine homeostasis in trained women cannot be answered at this time, as no studies are available.

Compared to dynamic strength, there was no increase in isometric grip strength in any group after the training intervention. On the contrary, a significant increase was observed in all groups at the end of the first phase, despite this phase being a control period without RT. The adaptation effects were more likely due to learning effects, as no RT was performed at this time. Surprisingly, grip strength did not improve during the subsequent intervention period, although most of the exercises undertaken require a strong grip, e.g. lateral flexion, barbell row and lat pull, which could have yielded improvements in grip strength. Similar to the data presented here, RT on machines for 12 weeks and three training sessions per week had no effect on grip strength [58]. Interestingly, grip strength is an important predictor of muscle status [59] and it is regularly employed to estimate the risk of all-cause mortality in the elderly [60]. However, if total body strength, but not grip strength, can be improved by RT with free weights, the relationship between grip strength, muscle status and therefore mortality may need re-examination.

Unlike muscular strength, significant differences between PreMeno and PostMeno women could be identified for FFM and MM. Significant curve progression was only observed in the PreMeno MI-RT group. A negative trend was also observed in the PostMeno LI-RT group. Compared to the PostMeno MI-RT group, MM decreased by -1.8 ± 2.0 kg between T0 and T2 (Table 3). Although there is no significant difference between PostMeno MI-RT and PostMeno LI-RT, the first indices suggest that this trend could not be observed in the PostMeno MI-RT group (-0.4 ± 2.4 kg). These results are in line with the observations of Karaaslan et al., who observed a significant decrease in lean body mass despite a 12-week intervention with 4 low intensity (40–50% 1-RM) training sessions per week. This result was not observed in the higher-intensity training group (70–80%) [56].

Training volume may also influence muscle growth in PostMeno women. Several attempts have been made to compare low-volume and high-volume training in RT research with elderly women [52, 55, 61–66]. For example, the results of Oliveira et al. showed that a higher training volume per week induced greater muscle growth in PostMeno women than low-volume training [61]. Both intervention groups performed machine-based training at an intensity of 80% 1-RM over 12 weeks with 3 training sessions each. The high-volume group had a total of 15 sets per exercise per week from week 3, whereas the low-volume group had only nine sets per week per exercise [61]. Radaelli et al. reported significantly greater quadriceps growth after 20 weeks of high-volume resistance training (6 sets per exercise per week) compared to low-volume resistance training (2 sets per exercise per week) [63]. Interestingly, similar to other studies [62, 64, 65], the authors reported training volume in terms of sets per exercise per week. However, very often the actual training volume per muscle group is higher than reported because training protocols include, for example, leg press exercises as well as leg extension or leg curl exercises. In Radaelli et al. [63], for instance, the high-volume group actually performed 12 sets of direct quadriceps training whereas the low-volume group performed 4 sets of direct quadriceps training, giving a more accurate picture of training volume distribution. Consequently, in both studies [61, 63], the high-volume groups performed more training sets per week per muscle group than the PostMeno LI-RT (six sets per week) and the PostMeno MI-RT (eight sets per week).

Importantly, not all studies found significant differences in favor of high-volume interventions compared to low-volume interventions for muscle growth [62, 64, 65]. However, in another study by Radaelli et al., the authors reported larger effect sizes after 6 weeks of training in high-volume protocols (12 sets of quadriceps training per week) compared to low-volume protocols (4 sets of quadriceps training per week) in the VL (effect size (ES) = 0. 33 vs. ES = 0.21), RF (ES = 0.28 vs. ES = 0.13), vastus medialis (ES = 0.37 vs. ES = 0.11), vastus intermedius (ES = 0.20 vs. ES = 0.14) and total quadriceps (ES = 0.45 vs. ES = 0.21) [64]. Similarly, Cunha and colleagues reported comparable significant increases in lean soft tissue between high-volume training (9 sets per exercise per week) and lower-volume training (3 sets per exercise per week), although slightly higher percentage changes (calculated by the authors of the present study) are observed in favor of high-volume training (appendicular lean soft tissue: 6.2% vs. 6.9%; upper limb lean soft tissue: 7.8% vs., 8.8%; lower limb lean soft tissue: 5.6% vs., 6.3%) [65]. Both groups performed exercises such as chess press, leg press, knee extension and leg curl. In common with previous studies, training volume was not reported for each muscle group, leading to the assumption that more volume was executed per muscle group than reported. Therefore, it can be speculated that PostMeno women require higher training volumes to induce greater muscle hypertrophic adaptations. Future studies should aim at longer intervention studies comparing higher training volumes with free weights (> 10 sets per muscle group per week) and lower training volumes (< 10 sets per muscle group per week).

Kang and colleagues also observed significant muscle growth with RT after 12 weeks [58]. Similar to de Oliveira, three training sessions with three sets per exercise were performed. In contrast to de Oliveria and the conducted intervention, a total of seven exercises were performed. The intensity of the exercises ranged from 55 to 65% 1-RM [58]. Consequently, it can be assumed that hypertrophy can be also induced with a higher training volume with moderate-intensity. In contrast to the BIA results as an indirect method of measuring MM, muscle thickness measurements showed a significant increase in RF over time, but no group differences. Moreover, in VL a trend over time (p = .050) and different curve progressions (p = .058) could be detected between the PreMeno MI-RT and PostMeno LI-RT groups. These results suggest that MI-RT results in superior adaptations in terms of muscle thickness compared to LI-RT. This is in line with the BIA results. However, there are no comparable data from previous studies.

FM decreased significantly only in the PreMeno MI-RT group. The results of both PostMeno groups do not confirm the observations of previous studies. Both Kang et al. [58] and Delshad et al. [67] observed a significant decrease in body fat after 12 weeks of RT. Similar to the effects on MM, training frequency and volume also play a decisive role in FM. This assumption can be supported by the results of Rodrigues et al., who found that FM did not decrease in a 12-week intervention study with two training sessions per week, including RT and endurance exercises [68]. The intensity of the training was controlled by a subjective Effort Perception Scale, which kept the intensity between 13 and 15 [68]. Therefore, both the intensities and frequencies of the resistance training and endurance parts were presumably too weak to activate fat metabolism.

### Limitations

Besides the important new findings, this study also has some limitations. One important factor is the small sample size of the individual training groups, so the observations must be regarded as preliminary evidence. Although there was a high level of interest in this study among women in this age group, almost 15% of those interested had to be excluded before the study started because of lack of mobility. In addition, training sessions were quickly cancelled for family reasons. Nevertheless, all participants reported that they enjoyed free weight training and were able to manage their daily lives better.

Another limitation of this study is the documentation of diet during the intervention. It was not possible to monitor the diet of all participants with a food diary over the entire duration. Therefore, only the protein and carbohydrate intakes immediately after exercise were ensured in order to stimulate protein biosynthesis as quickly as possible and to promote recovery in the best possible way. Even though the participants were instructed not to change their diet, this could also affect the results. Furthermore, it is not possible to say whether the participants had a sufficient protein intake to promote muscular adaptation in the best possible way. Therefore, future studies in this population should consider the effects of diet, and especially protein intake. Due to the hormonal changes and body composition in post-menopausal women, protein intake should be calculated based on FFM to ensure the best possible feasibility.

We additionally collected saliva and blood samples from premenopausal women once at T0. Although E2 and P are highest during the luteal phase when we collected our samples, its rather speculative whether the highest concentrations of E2 and P were obtained given that E2 and P vary during the menstrual cycle and the luteal phase itself. [69].

## Conclusion

In conclusion, the results of this study show that free weight RT is generally safe and effective for middle-aged women. Free weight, moderate-intensity RT twice a week leads to an increase in 1-RM squat and bench press performance, as well as an increase in muscle mass and a decrease in fat mass in pre-menopausal middle-aged women. In post-menopausal women, RT induces an increase in dynamic strength but not in muscle mass, which can be induced by RT irrespective of intensity. However, there is some evidence that a higher intensity led to better effects on muscle mass. It seems that the general recommendations for anaerobic exercises, such as resistance training, do not lead to increases in muscle mass and decreases in fat mass in post-menopausal women [24, 70]. It appears that post-menopausal women require more than two training sessions and more than six to eight sets per muscle group/week, as well an intensities of more than 50% 1-RM elicit changes in body composition. These hypotheses are supported by two meta-analyses of dose-response relationships in elderly [71, 72].

### Electronic supplementary material

Below is the link to the electronic supplementary material.

Supplementary Material 1

## Data Availability

The raw data of the participants can be requested from the corresponding authors if required. All data was encrypted so that it cannot be traced back to individual persons.
