# Supplementary material for: Resistance training alters body composition in middle-aged women depending on menopause - A 20-week control trial
Source: BMC Womens Health. 2023 Oct 6;23:526. doi: 10.1186/s12905-023-02671-y (PMC10559623; doi:10.1186/s12905-023-02671-y)
Supplement: Supplementary file 1 — Supplementary Material 1 [file 12905_2023_2671_MOESM1_ESM.docx]

**Supplemental Material**

Table: Carbohydrate protein meals after training

|  | Carbohydrate (g) | Protein (g) | Fat (g) | Calories (kcal) |
| --- | --- | --- | --- | --- |
| 52g Protein*-45g carbohydrate**-shake | 45.5 | 38.4 | 8.2 | 374.6 |
| 76g White bread with 100g milk cheese | 35.9 | 36.4 | 1.9 | 312.0 |
| 250g low fat curd cheese with 250ml grape juice | 50.9 | 30.7 | 0.5 | 340.0 |

**=LSP Premium Whey Protein (LSP® Sporternährung GmbH, Bonn), **= maltodextrin (LSP Maltodextrin (LSP® Sporternährung GmbH, Bonn, Germany).*
